# Publication trends of shared decision making in 15 high impact medical journals: a full-text review with bibliometric analysis

**DOI:** 10.1186/1472-6947-14-71

**Published:** 2014-08-09

**Authors:** Xavier Blanc, Tinh-Hai Collet, Reto Auer, Roland Fischer, Isabella Locatelli, Pablo Iriarte, Jan Krause, France Légaré, Jacques Cornuz

**Affiliations:** 1 Department of Ambulatory Care and Community Medicine, University of Lausanne, Rue du Bugnon 44, 1011 Lausanne, Switzerland; 2 Service of Endocrinology, Diabetes and Metabolism, University Hospital of Lausanne, Lausanne, Switzerland; 3 Medicine Faculty Library, University Hospital of Lausanne, Lausanne, Switzerland; 4 Chaire de recherche du Canada, Implantation de la prise de décision partagée dans les soins primaires, Département de médecine familiale et de médecine d’urgence, Université Laval, Centre Hospitalier Universitaire de Québec, Québec, Canada

**Keywords:** Shared decision making, Bibliometric analysis, Decision making, Full text search, Review, Information storage and retrieval, PubMed, Text mining

## Abstract

**Background:**

Shared Decision Making (SDM) is increasingly advocated as a model for medical decision making. However, there is still low use of SDM in clinical practice. High impact factor journals might represent an efficient way for its dissemination. We aimed to identify and characterize publication trends of SDM in 15 high impact medical journals.

**Methods:**

We selected the 15 general and internal medicine journals with the highest impact factor publishing original articles, letters and editorials. We retrieved publications from 1996 to 2011 through the full-text search function on each journal website and abstracted bibliometric data. We included publications of any type containing the phrase “shared decision making” or five other variants in their abstract or full text. These were referred to as SDM publications. A polynomial Poisson regression model with logarithmic link function was used to assess the evolution across the period of the number of SDM publications according to publication characteristics.

**Results:**

We identified 1285 SDM publications out of 229,179 publications in 15 journals from 1996 to 2011. The absolute number of SDM publications by journal ranged from 2 to 273 over 16 years. SDM publications increased both in absolute and relative numbers per year, from 46 (0.32% relative to all publications from the 15 journals) in 1996 to 165 (1.17%) in 2011. This growth was exponential (P < 0.01). We found fewer research publications (465, 36.2% of all SDM publications) than non-research publications, which included non-systematic reviews, letters, and editorials. The increase of research publications across time was linear. Full-text search retrieved ten times more SDM publications than a similar PubMed search (1285 vs. 119 respectively).

**Conclusion:**

This review in full-text showed that SDM publications increased exponentially in major medical journals from 1996 to 2011. This growth might reflect an increased dissemination of the SDM concept to the medical community.

## Background

Shared decision making (SDM) is increasingly advocated as a model of best practice for decision making in the medical encounter [1-3]. SDM has been defined as a process by which healthcare choices are made jointly by the physician and the patient [4]. Since the mid-1990s, an increasing number of publications on SDM have been published not only in the field of medicine, but also in sociology, psychology, economics, and ethics [5]. The Patient Protection and Affordable Care Act, signed into the law of the United States in March 2010, contains provisions aimed at encouraging the use of SDM, which represents an important entry point for SDM into public policy [6-8]. Despite these positive developments, the use of SDM remains low due to many barriers blocking its full implementation in clinical practice [9-11].

Research publications whose main topic is not necessarily SDM may not mention the phrase “shared decision making” in the title or the abstract, but only in the discussion. Editorials, reviews or debates, among other publication types, do not have abstracts but have a potential impact on the reader. Moreover, not every single phrase is indexed in PubMed. All these publications might then be missed out by a systematic search of titles, abstracts and keywords on PubMed [12,13]. In contrast, a search of full-text articles would enhance the publication retrieval [14].

Within the last years, SDM increasingly appears in editorials and articles’ discussions in high impact medical journals. However, no reliable data can support this assertion. Such journals play a major role in the dissemination of new medical evidence [15,16]. The frequency of SDM in top journals in general internal medicine may reflect its importance in the medical community. Therefore, we conducted a full-text search followed by bibliometric analysis to identify and characterize publication trends in SDM in 15 major general internal medicine journals.

## Methods

We selected the 15 journals with the highest 5-year impact factor in 2010 based on the ISI Web of Knowledge Journal Citation Reports, [17,18] in the category “medicine, general and internal”, that already existed in 1996 and regularly published original articles, letters and editorials (Additional file 1: Table S1). We did not include journals publishing only reviews, like *Cochrane Database of Systematic Reviews*, in order to cover various types of publications, such as research publications and editorials, and to reduce heterogeneity between journals.

### Search method and publication selection

We retrieved publications through the full-text search function on each journal website, usually located on the “advanced search” web page or on the publisher website if not available. We typed chosen keywords as a phrase in double quotes in the search box. We used this search method, defined as website full-text search, because it allows finding publications with a search term not necessarily in the title or abstract, but in the article sections in full text. This is not possible through a PubMed search as full texts are not available on the PubMed search engine. We included publications of any type, with the exception of cover pages, tables of content and indexes (of authors, keywords, etc.).

To identify publications relating to SDM, referred to as SDM publications, we first chose the phrases “shared decision making” and “informed decision making” because they are the most often encountered in medical literature [19]. To improve search sensitivity, we added variants with the word “medical” (“shared medical decision making”, “informed medical decision making”). We also studied combinations of those two phrases (“informed and shared decision making”, “informed shared decision making” [4]).

We included publications released between January 1996 and December 2011, because the concept of SDM began to appear significantly in medical literature since the mid-1990s [5,19]. Moreover, electronic publications only became widely available on journal websites around 1995–97 due to changes in the publishing framework that permitted the use of automated search engines [20].

### Abstraction of bibliometric data

We abstracted the following bibliometric data from the full text of included SDM publications on a prespecified electronic form (EpiData Software, version 3.1, EpiData Association, Denmark): journal, publication year, publication type, publication topic, and exact phrase relating to SDM. Publications assessing decision aids were distinguished from those that did not. We categorized publications into 9 types adapted from the indexing methods of PubMed, Embase, and previous bibliometric studies [21-23]. We grouped publications into 9 clinical topics according to chapter titles of a textbook on SDM [24] and 4 non-clinical topics, derived from a study [21] and iterative processes. We considered topics as non-clinical when publications did not address any clinical specialty. We used the definition of the Cochrane Review on decision aids: “interventions designed to help people make specific and deliberative choices among options (including the *status quo*) by providing (at the minimum) information on the options and outcomes relevant to a person’s health status and implicit methods to clarify values” [25]. Two authors (X.B. and R.F.) carried out single data abstraction. To measure reliability of abstraction in our prespecified form, both authors independently abstracted a random selection of publications to calculate agreement rate and Cohen kappa [26].

### Validation of website full-text search

This novel search method relied on journal/publisher websites whose search strategies were not explicit, in contrast to PubMed searches. To compare our method results with a validation dataset, we contacted the editorial board of each journal to request authorisation to obtain all published materials since 1996. After receiving authorisation from the journals (n = 6/15), we collected published materials and launched an automated search script (Python Software, version 2.6, Python Software Foundation, Wolfeboro Falls, NH, USA). We used the same six phrases relating to SDM for publication retrieval. This text retrieval method on a locally stored full-text corpus was defined as downloaded full-text search [27]. We assessed the numbers of retrieved publications and the reliability between the website and downloaded full-text searches.

To assess the performance of the website full-text search, we compared it with a PubMed search using a similar strategy, i.e. using the same six phrases relating to SDM in the 15 journals between 1996 and 2011. We typed the six phrases in double quotes in the search box, so that the PubMed search looked for the exact phrase in all fields, such as the titles and abstracts, but also indexed terms and other metadata recorded by PubMed database.

### Statistical analyses

We first conducted simple descriptive statistics of publications in each journal. We compared the overall number of publications with the number of SDM publications from 1996 to 2011. We assessed descriptive statistics of bibliometric data of SDM publications. We used a polynomial Poisson regression model with logarithmic link function [28,29] to assess the evolution of the number of SDM publications, the dependent variable, according to different publication characteristics. These covariables were abstracted from the selected publications: publication type (dichotomized into research and non-research publications), topic (clinical and non-clinical), and variant of SDM phrase. A Poisson regression model with offset [30] was used to assess the evolution of the percentage of SDM publications with respect to the overall number of publications in a year. We used the R statistical computation and graphics package (version 2.12.1, GNU Project, University of Auckland, New Zealand) and considered P < 0.05 as significant.

## Results

**Figure 1:**
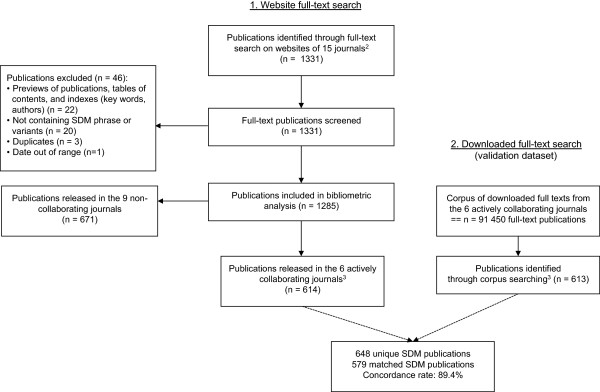
**Flow diagram of publications evaluated for inclusion in bibliometric analysis**^**1 **^**and comparison of both search methods. **^1^Adapted from the flow diagram in the PRISMA Statement. ^2^CMAJ: publications not available in full text for years 1996-1999, identified through PubMed Central. ^3^Search limited to years 1996-2010 due to technical reasons.

The results of the full-text search on the journal websites are shown in Figure 1 (adapted from PRISMA [31]). We retrieved 1331 publications out of a total of 229 179 released between 1996 and 2011 in the 15 journals with the highest 5-year impact factor in 2010. We screened the full texts and included 1285 publications in bibliometric analysis, referred to as SDM publications. The main reason for exclusion was that publications were not proper articles but previews of articles, tables of contents or indexes. Twenty other publications were excluded because they were not containing the SDM phrase or variant.

**Figure 2:**
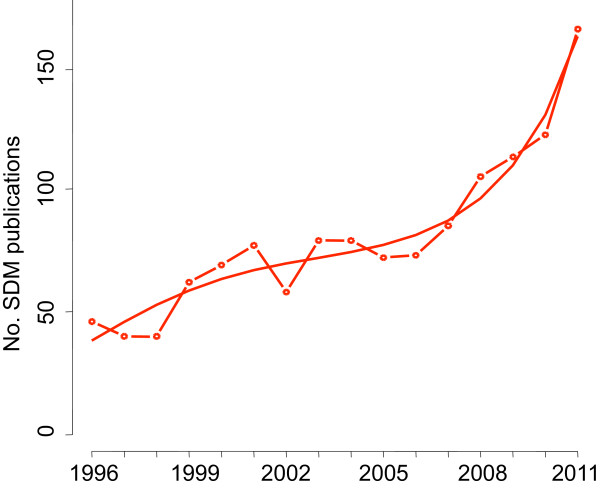
**Absolute numbers of SDM publications over time.** The curve was estimated in a Poisson model; with the number of SDM publications in a year as a dependent variable (N) and year since 1996 as an independent variable (t). N = exp(3.64 + 0.21 ∗ t - 0.02 ∗ t^2^ + 0.001 ∗ t^3^). All coefficients P < 0.01.

**Figure 3:**
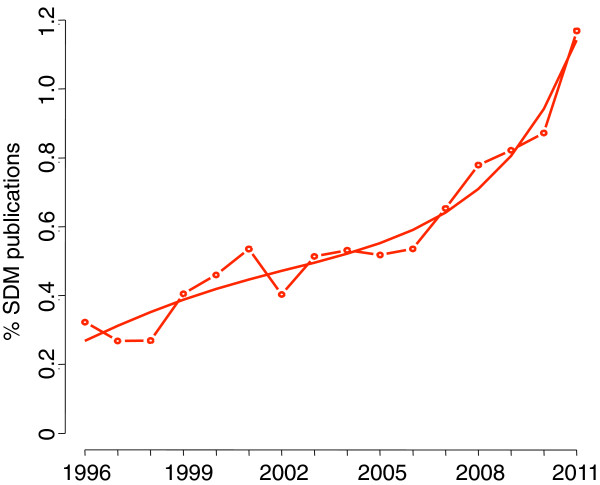
**Relative numbers of SDM publications over time.** The curve was estimated in a Poisson model; with the percentage of SDM publications over the total of the year as a dependent variable (100 ∗ N/Total) and year since 1996 as an independent variable (t). 100 ∗ N/Total = 100 ∗ exp(-5.92 + 0.16 ∗ t - 0.02 ∗ t^2^ + 0.0008 ∗ t^3^). All coefficients P < 0.05, except for t^2^ coefficient P < 0.10.

The number of SDM publications varied by journal from 2 in *Annals of Medicine* to 273 in the *BMJ* over 16 years (Table 1). The number of SDM publications relative to the total number of publications by journal ranged from 0.09% in *The Lancet* to 4.99% in *Journal of General Internal Medicine*. SDM publications increased both in absolute and relative numbers per year, from 46 (0.32%) in 1996 to 165 (1.17%) in 2011. In a Poisson model (see equations in figures footnotes), this increase in absolute numbers was significant (all coefficients P < 0.01) and exponential (Figure 2). In relative numbers, the growth was similar (Figure 3) and also significant (all coefficients P < 0.05, except for t^2^ coefficient P < 0.10).The number of SDM publications was much larger in 2011 than during previous years (Figure 2). Considering this value as a possible outlier, we performed a post-hoc sensitivity analysis without year 2011 and observed a similar pattern.

**Table 1:** Number of SDM publications in 15 journals in 1996–2011*

**Journal**	**Total no publications**	**No SDM publications**	**%**
British Medical Journal	61 903	273	0.44
Journal of General Internal Medicine	4267	213	4.99
Journal of the American Medical Association	32 868	206	0.63
Annals of Internal Medicine	11 950	117	0.98
Archives of Internal Medicine	8388	105	1.25
American Journal of Preventive Medicine**	3442	82	2.38
Canadian Medical Association Journal	18 686	71	0.38
Journal of Pain and Symptom Management	3814	48	1.26
The New England Journal of Medicine	19 778	48	0.24
The Lancet	45 325	41	0.09
Preventive Medicine	3368	29	0.86
The American Journal of Medicine	6780	28	0.41
Mayo Clinic Proceedings	4810	19	0.40
Journal of Internal Medicine	2544	3	0.12
Annals of Medicine	1256	2	0.16
**Overall (15 journals)**	**229 179**	**1285**	**0.56**

*Abbreviations [for all tables]:SDM* Shared Decision Making, *SDM publications* publications of any type containing in their full text the phrase “shared decision making” or five other variants.

*Journals ordered by absolute number of SDM publications.

**Publications not available for years 1996–1997 for technical reasons due to a change of publisher in 1998.

**Figure 4:**
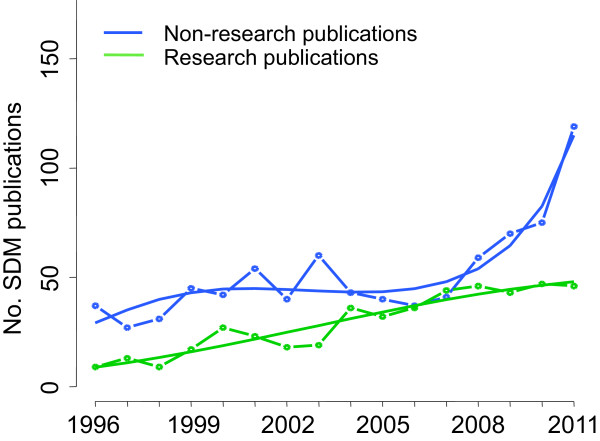
**Absolute numbers of SDM publications over time according to publication type.** The curve was estimated in a Poisson model; with the number of non-research publications in a year and the number of research publications in a year as dependent variables (N_0_ and N_1_, respectively) and year since 1996 as an independent variable (t). N_0_ = exp(3.37 + 0.22 ∗ t - 0.04 ∗ t^2^ + 0.002 ∗ t^3^). N_1_ = exp(2.17 + 0.22 ∗ t - 0.01 ∗ t^2^). All coefficients P ≤ 0.001.

Of all 1285 SDM publications, there were fewer research publications (465, 36.2%) than non-research publications (Table 2). The dynamics of the growth was also significantly different: while the number of research publications increased almost linearly, from 9 in 1996 to 46 in 2011, the number of non-research publications showed an acceleration in the second part of the time period (Figure 4).

**Table 2:** Characteristics of SDM publications

	**No**	**%**
Publication type*		
Research publications	465	36.2
Observational study (case report/series, cohort study, case–control study, …)	287	22.3
Interventional study (randomized controlled trial, before-after study, …)	97	7.5
Systematic review with or without meta-analysis	43	3.3
Guideline, consensus publication	38	3.0
Non-research publications	820	63.8
Non-systematic review (narrative, clinical case, debate, …)	379	29.5
Letter, comment, book review	250	19.5
Editorial	145	11.3
Conference publication (abstract, paper, review)	40	3.1
Other (animal study, biology study, government report, unclear study type, combination study design, …)	6	0.5
**Topic**		
Clinical	645	50.2
Gynaecology & Obstetrics (including prenatal testing and other foetal issues)	118	9.2
End-of-life care & Cardiopulmonary Resuscitation	114	8.9
Cardiovascular system (including diabetes, CV diseases prevention, CV risks management)	101	7.9
Urology (including prostate issues)	86	6.7
Gastroenterology (including colorectal cancer)	54	4.2
Surgery, Orthopaedics & Rheumatology (including low back pain and osteoporosis)	41	3.2
Psychiatry/Mental Health (including addiction medicine)	28	2.2
Paediatrics & Genetics	26	2.0
Other**	77	6.0
Non-clinical	640	49.8
Medical education^†^ (including communication skills and medical management)	203	15.8
Decision making (including shared decision making and ethics)	199	15.5
Patient care management^††^ (including patient education)	119	9.3
Public health^‡^	119	9.3
**Publications assessing decision aids**	60	4.6
**Overall**	**1285**	**100.0**

*Categories adapted from indexing methods of PubMed, Embase, and bibliometric studies [21-23].

**Other clinical topics: anaesthesiology, complementary and alternative medicines, emergency medicine, endocrinology, ENT, infectious diseases, intensive care, nephrology, neurology, oncology, ophthalmology, pathology, pharmacology, radiology, respiratory system.

^†^Education, training programs, and courses in various fields and disciplines in medicine.

^††^Generating, planning, organising, and administering medical and nursing care and services for patients.

^‡^Branch of medicine concerned with the prevention and control of disease and disability, and the promotion of physical and mental health of the population on the international, national, state, or municipal level, including health care delivery and health promotion, e.g. physical activity, smoking management.

SDM was approached through a range of clinical topics in the included publications; gynaecology/obstetrics (118 publications, 9.2%), end-of-life care (114, 8.9%), and cardiovascular system (101, 7.9%) were the most frequent clinical topics (Table 2). We found no statistically significant difference between SDM publications addressing a clinical topic and those addressing a non-clinical topic (P > 0.30 both at baseline and across the period). The progression pattern of both was similar to the general trend (data not shown).

**Figure 5:**
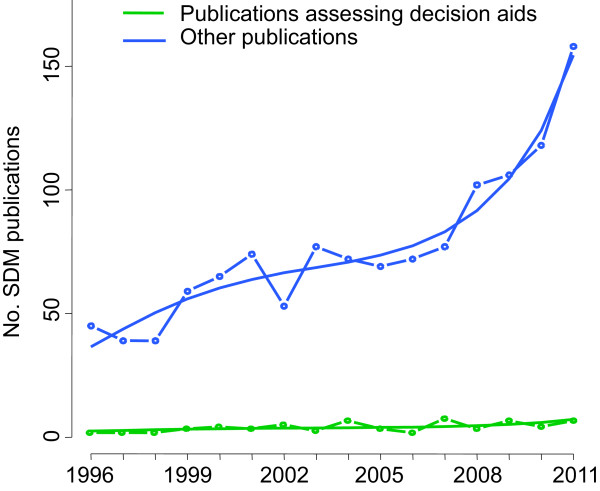
**Absolute numbers of SDM publications over time and publications assessing decision aids.** The curve was estimated in a Poisson model; with the number of other publications in a year and the number of publications assessing decision aids in a year as dependent variables (N_0_ and N_1_, respectively) and year since 1996 as an independent variable (t). N_0_ = exp(3.60 + 0.21 ∗ t - 0.02 ∗ t^2^ + 0.001 ∗ t^3^). N_1_ = exp(0.58 + 0.21 ∗ t - 0.02 ∗ t^2^ + 0.001 ∗ t^3^). All coefficients P < 0.01.

We found 60 publications assessing decision aids over 16 years (4.6% relative to all SDM publications), most of them randomized controlled trials (Table 2). These publications showed the same growth pattern as that of the other publications (Figure 5).

### Validation of website full-text search and data abstraction

The concordance rate was high (89.4%) between the downloaded and website full-text searches (Figure 1). Two publications were retrieved by the downloaded full-text search but not by the website full-text search. The reason was a defect in the automated Optical Character Recognition (OCR) of those publications. Both were research publications.

The website full-text search identified about ten times more publications than a systematic search through PubMed (1285 vs. 119). Out of 119 publications, 3 were missed out by the website full-text search; one because of unavailable data on the journal website for years 1996–97 and two were available on the journal website but the remote search engine failed to retrieve them for an unknown reason.

Two authors independently assessed a random selection of 173 publications out of the 1285 to measure the reliability of the dichotomized data of our prespecified form. The agreement rate and Cohen kappa between the two authors were as follows: for publication type, 95.4% and 0.89, respectively; for publication topic, 93.6% and 0.87, respectively; for publications assessing decision aids, 93.6% and 0.24, respectively.

## Discussion

We found a significant and exponential increase in the number of SDM publications in the 15 highest impact factor journals in general internal medicine between 1996 and 2011. Nevertheless, the number of SDM publications across the journals varied widely. When focusing on research publications, the growth was not exponential but remained mostly linear since 1996. We observed few publications assessing decision aids, however their growth pattern was similar to the general trend.

Several studies have explored the frequency of concepts in medical literature [5,21] or focused on research productivity [18,32]. Through a PubMed search of the term SDM, a previous study showed a sharp growth of the number of publications in the overall literature from 1996 to 2003 [5]. Our results are concordant with these findings. We also obtained relative numbers to make sure that the increase was not only due to the growing number of overall publications in the studied journals.

More recently, a bibliometric analysis assessed the quality as well as the volume of research in primary care between countries [32]. Our review in full-text, however, identified not only research publications but other publication types as well, such as editorials or narrative reviews, which could also have an impact on the readers. Moreover, we showed that the exponential increase of SDM observed in the last 16 years was mainly due to these types of publications, while research publications followed a linear trend during the same period.

Another study measured the distribution of topics in two major medical journals by disease categories and domains over a calendar year [21]. They found that the topic prevention was underrepresented in the journals in comparison to the importance of this topic to patients and public health. In our study, we observed a large variation in the number of SDM publications across the 15 journals studied. In relative numbers, the *Journal of General Internal Medicine* released fifty times more publications than the last journal in the top 15. This variation may be partially explained by differences in editorial choices, which were not studied here. Nevertheless, the trends were analysed according to various components. Breaking down the increase in SDM publications into bibliometric data brought some keys to better understand it.

To our knowledge combining techniques of review through full-text search and bibliometric analysis has not been reported before. Full-text search techniques have been used in biomedicine, [12] especially genomics, [33] but not yet in clinical medicine. This new combined method offers both search precision and simplicity of use. The sensitivity of detecting phrases in full-text publications is much higher than when limited to a classical PubMed search [13,34]. We validated the method of full-text search on journal websites through a good concordance rate when comparing with the validation dataset. Moreover, this method did not present the difficulties that we met in collecting the validation dataset from some uneasy publishers, a known issue in the scientific field [35]. Validity of our abstraction form was also gained through high kappa values for the different categories of bibliometric data.

### Limitations

Our study has some limitations. First, our study was limited to the 15 major medical journals chosen according to their impact factor. Despite the widespread use of the impact factor metric, [36] it has inherent limitations [37-39]. We considered an alternative journal selection process based on journal circulation as a reflection of readership [40]. Nevertheless, circulation counts are affected by the current increase in readers accessing journals online.

Second, categorising publication types from 15 different journals was limited by our a priori criteria as each journal has its own indexing method. Our category list was developed internally, inspired by PubMed and Embase classifications, yet we reached a high degree of agreement between both reviewers (agreement rate 95.4%, Cohen kappa 0.89). The discrepancy between agreement rate (93.6%) and Cohen kappa (0.24) on publications assessing decision aids could be explained by the imbalance of the 2×2 table and the low number of publications reporting on decision aid tools [41,42].

Third, we did not divide papers into those where the concept of SDM was the primary topic and those where the concept was briefly mentioned. Our aim was to perform a scoping review of the concept of SDM in the selected medical journals. Future, more detailed studies could aim at better understanding if the concept was used as the primary topic or if authors just mentioned it.

Fourth, two journals did not exist before 1996 and were therefore excluded from our analyses. These were two open access journals (*PLOS Medicine* and the *Annals of Family Medicine*). We cannot determine if the trends in SDM would have been different in open access journals. Future studies might aim at determining if the trends in SDM are different between open access and other journals.

Finally, if we showed an increase in the number of SDM publications, we did not assess to which extent the medical community reads them. Therefore, the impact of this increase remains unknown. A future study could alternatively focus on the number of citations per SDM publication compared to citations of other publications [43].

The growth in SDM publications found in our study nevertheless supports the call of experts in the field for the medical community to implement SDM in practice [2,3,44,45]. Some of these calls have been published in major medical journals. These journals are thought to have a large impact on a vast population of physicians [15,46]. More than three years after the enactment of the Patient Protection and Affordable Care Act, there are concerns that the SDM model has not been promoted enough [7,8]. Addressing SDM in major journals is therefore more important than ever, as it could be an efficient way to disseminate it among the medical community. Moreover, our study may capture the transition of interest and advocacy for SDM from experts to clinicians and policy makers [8,47].

SDM has been called to ensure evidence-based patient choice, especially for equipoise situation [48]. Even though some issues have been raised threatening the compatibility between EBM and SDM, such as practice or financial incentives to achieve quality standards, both approaches are now clearly justified to promote efficient care by integrating patient autonomy [49].

## Conclusion

This study shows that SDM publications increased exponentially in major medical journals from 1996–2011. This growth might reflect an increased dissemination of the SDM concept to the medical community. We explored a novel methodology by combining review through full-text search with bibliometric analysis. The methodology permitted a thorough retrieval of SDM publications and a precise analysis of the dissemination of SDM in medical literature.

## Competing interests

The authors declare that they have no competing interests.

## Authors’ contributions

RA, THC, XB were involved in the design, implementation, and analysis of the study. FL and JC were involved in the design and analysis of the study. RF and XB abstracted data. IL did the statistical analysis. PI and JK were responsible for the downloaded full-text search. XB drafted the manuscript. All authors were involved in the interpretation of the findings. All authors critically read and approved the final manuscript.

## Pre-publication history

The pre-publication history for this paper can be accessed here:

http://www.biomedcentral.com/1472-6947/14/71/prepub

## Supplementary Material

Additional file 1: Table S1 Selection of 15 journals in general internal medicine. Click here for file
