# Supplementary material for: Publication trends of shared decision making in 15 high impact medical journals: a full-text review with bibliometric analysis
Source: BMC Med Inform Decis Mak. 2014 Aug 9;14:71. doi: 10.1186/1472-6947-14-71 (PMC4136407; doi:10.1186/1472-6947-14-71)
Supplement: Additional file 1: Table S1 — Selection of 15 journals in general internal medicine. [file 1472-6947-14-71-S1.pdf]

Additional Table S1. Selection of 15 journals in general internal medicine

Rank	Journal	5-year impact factor (ISI* 2010)	Reason of exclusion
1	The New England Journal of Medicine	52.36	
2	The Lancet	32.50	
3	Journal of the American Medical Association	29.31	
4	Annals of Internal Medicine	16.76	
Excl.	Plos Medicine	14.97	Start after 1996
5	British Medical Journal	11.94	
Excl.	The Annual Review of Medicine	10.89	Only reviews
6	Archives of Internal Medicine	10.39	
7	Canadian Medical Association Journal	8.07	
Excl.	Cochrane Database of Systematic Reviews	6.35	Only reviews
Excl.	Medicine	5.91	Only reviews
8	Journal of Internal Medicine	5.61	
9	The American Journal of Medicine	5.11	
10	Mayo Clinic Proceedings	5.03	
11	American Journal of Preventive Medicine	4.99	
Excl.	The Annals of Family Medicine	4.97	Start after 1996
12	Annals of Medicine	4.67	
13	Journal of General Internal Medicine	3.62	
14	Preventive Medicine	3.52	
Excl.	British Medical Bulletin	3.37	Only reviews
15	Journal of Pain and Symptom Management	3.19	

* ISI Web of Knowledge Journal Citation Reports, Thomson Reuters, 2010. Available from: http://admin-apps.webofknowledge.com/JCR/JCR.
